# Language and Cognition in Gaelic-English Young Adult Bilingual Speakers: A Positive Effect of School Immersion Program on Attentional and Grammatical Skills

**DOI:** 10.3389/fpsyg.2020.570587

**Published:** 2020-10-20

**Authors:** Maria Garraffa, Mateo Obregon, Bernadette O’Rourke, Antonella Sorace

**Affiliations:** ^1^LangLifeLab, Department of Psychology, School of Social Sciences, Heriot-Watt University, Edinburgh, United Kingdom; ^2^Institute for Language, Cognition and Computation, School of Informatics, The University of Edinburgh, Edinburgh, United Kingdom; ^3^School of Modern Languages and Cultures, University of Glasgow, Glasgow, United Kingdom; ^4^School of Philosophy, Psychology and Language Sciences, The University of Edinburgh, Edinburgh, United Kingdom

**Keywords:** bilingualism, school immersion, minority languages, grammar, executive functions, Gaelic, relative clauses

## Abstract

The present study investigates linguistics and cognitive effects of bilingualism with a minority language acquired through school medium education. If bilingualism has an effect on cognition and language abilities, regardless of language prestige or opportunities of use, young adult Gaelic-English speakers attending Gaelic medium education (GME) could have an advantage on linguistic and cognitive tasks targeting executive functions. These will be reported, compared to monolingual speakers living in the same area. Furthermore, this study investigates whether there is a difference in Home Speakers of Gaelic (speakers who had acquired the language at home) compared to New Speakers of this language, i.e., whether an immersive context-as the one offered in medium education- compensates for not being native. A group of 23 monolingual English young adult speakers was compared with a group of 25 bilingual speakers attending a GME school since 5 years old. Participants were tested on comprehension of a set of sentences with incremental complexity in English, on their capacity to inhibit a distractor using the Test of Everyday attention (TEA) and on their performance in a Digit Span task. A tendency for a better performance on more complex linguistics and cognitive tasks was reported in bilinguals compared to monolinguals with a further advantage for New Speakers compared to Home Speakers. The study supports the idea that being bilingual in a minority language is as beneficial as speaking any other combination of languages. An immersive context of acquisition can be a good ground for developing advantages on both linguistics and cognitive tasks, with a further advantage for New speakers of the language.

## Introduction

There has been an increased interest in bilingualism research due to the significant rise of the number of speakers of more than one language. To date, estimates indicate that at least half of the world’s population is bilingual, and this number is on the rise (Grosjean, 2010; Marian and Shook, 2012). However, while bilingualism covers an entire spectrum of possible language combinations, the number of studies on minority languages such as Gaelic in Scotland is still relatively scarce, despite the fact that many of these languages are under threat because intergenerational transmission no longer enables their maintenance and survival (Fishman, 1991; McLeod et al., 2010; Gorter and Cenoz, 2011; Hornsby and Agarin, 2012). One of the main reasons for the lack of intergenerational transmission of minority languages is their low prestige status, and consequent perceived lack of usefulness, compared to globally spoken languages. This discrepancy is also mirrored in bilingualism research, which has largely focused on widely spoken languages (but see Gorter and Cenoz, 2011; Garraffa et al., 2015; for reviews of studies on minority languages).

The present study contributes to bridging the gap in research on bilingualism in minority language speakers by investigating the linguistic and cognitive skills of Gaelic-English young adults attending a Gaelic-medium school (“new speakers,” who have no Gaelic at home and have acquired Gaelic at school) and comparing monolingual English speakers living in the same area with both native speakers of Gaelic and new speakers of Gaelic.

### Bilingualism in the Scottish Gaelic Context

**FIGURE 1:**
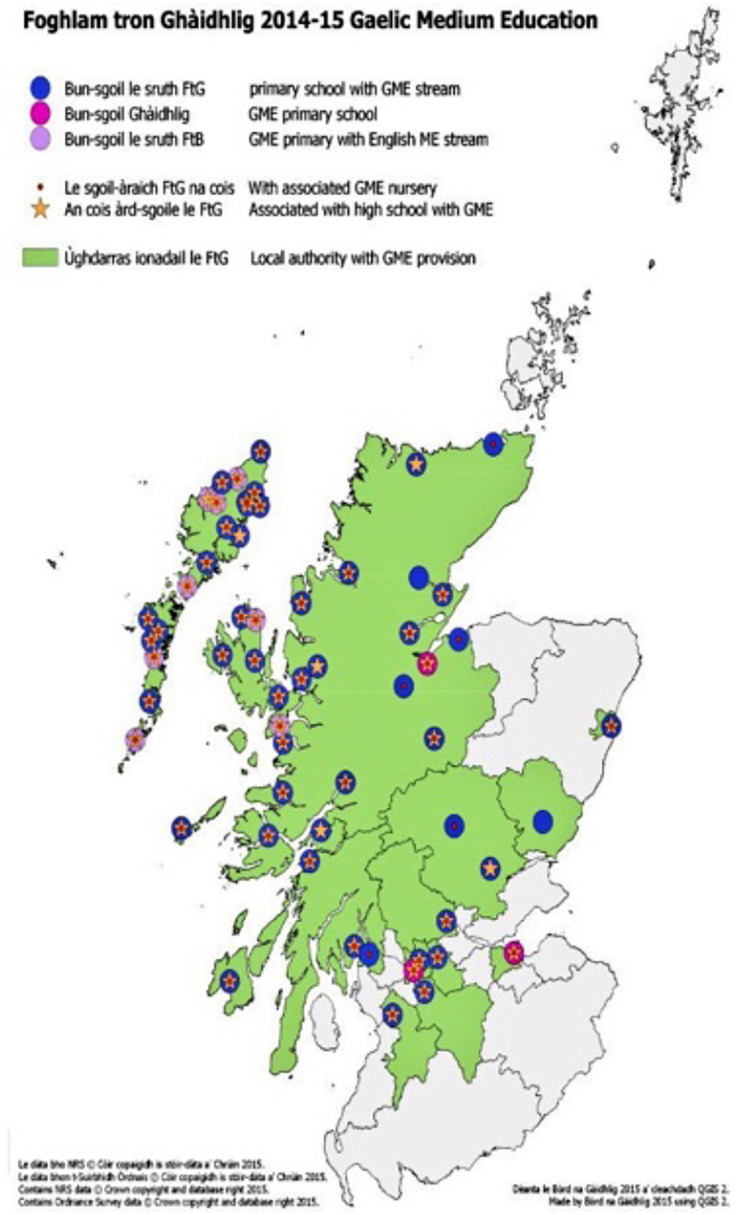
Map of the locations with Gaelic Medium Education in Scotland.

Bilingualism with minority languages differs from bilingualism with more global languages in various respects, including the quality and quantity of input received by children, the attitudes toward the language and its social status, and the motivations for bilingual upbringing, often linked to heritage values. Therefore, the number of speakers of many minority languages has been reported to be systematically decreasing (McLeod et al., 2010). Indeed, studies have found that the intergenerational transmission of Gaelic has been steadily declining for decades, the tendency being even stronger outside the Highlands (see National Language Plan, Bòrd na Gàidhlig, 2007–2012). This decrease is further strengthened by the fact that native speakers of Gaelic tend to increasingly use English within the family, even when the language is learned by the younger generation (McLeod et al., 2010). Implications are threefold. First, not only may Gaelic learners get less exposure to Gaelic, but also the input may be less varied (due to a narrower range of speakers and few dialectal variations; see Houston and Jusczyk, 2000), may be affected by language attrition, or may be produced by people who learnt Gaelic as a second language. Second, there is a marked decrease in the number of speakers whose first language is Gaelic. Third, speakers’ confidence when speaking Gaelic tends to be low due to lack of perceived usefulness in speaking the language. Furthermore, native Gaelic speakers are increasingly refraining from speaking and transmitting Gaelic to their children, due to negative attitudes discouraging its use. In this context, the approval of the Gaelic Language (Scotland) Act 2005 marks a pivotal moment in the history of Gaelic, since for the first time Gaelic has been included in a political agenda and considered equal to English. The Gaelic language plan emphasizes the fundamental role of Gaelic medium education (GME) schools in passing on and preserving Gaelic (Bòrd na Gàidhlig, 2007, 2012). These GME schools (Figure 1) are one of the pillars of the Gaelic revival strategy and are gaining popularity thanks to the high quality of education, the wish to preserve Scotland’s heritage, and the more widespread awareness of the benefits of bilingualism (O’Hanlon, 2015).

The basic idea behind these policies is that preservation of minority languages rests mainly on a new generation of young adult speakers. Specifically, these policies aim at boosting the number of Gaelic speakers by including young adult learners, in the hope that it will lead to a Reversal Language Shift (RLS) (Fishman, 1991). Over the last 30 years, many schools have developed GME programs, in which curricular contents are taught through the medium of Gaelic to both native speakers and new speakers (students with no home Gaelic background). This second category represents a crucial asset for the revitalization of the language. However, up to now there has been no research on the linguistic competence and overall cognitive effects of bilingualism in these “New Speakers” in comparison with native speakers of a minority language. The aim of this study is precisely to begin to provide empirical evidence about this population who have now been attending medium education programs for more than 10 years.

### Scottish Gaelic: An Overview

Scottish Gaelic (ScG) is a minority language spoken predominantly in the North West of Scotland and in some urban pockets in the central belt around Glasgow. The language belongs to the Goidelic branch of the Celtic family of Indo-European languages with no connection with the dominant language spoken in the area, English. This study is focusing on grammatical competence in bilingual Gaelic/English speakers. In the paragraph below we will present some features of Gaelic grammar aiming at describing the differences with English, the dominant language spoken in the environment of our participants.

Gaelic is primarily a Verb Subject Object (VSO) language, with considerable phonological, morphological and syntactic variation depending on factors, including dialects and the age of the speaker (Lamb, 2008; Adger, 2009a, b).

Gaelic has a system of cases (nominal, prepositional, possessive, and vocative), all morphologically marked on nouns. The nominal case is used for the citation form of a word. The genitive case is used to express a possessive relation between nouns, as in the nominal form *casan a’ bhùird* (the table’s legs). In the possessive forms only the noun denoting the possessor takes the genitive case. It is interesting to note that differently from English in these constructions Gaelic places an article only in front of the second noun. The vocative case is used when addressing a person, for example in a speech or in a letter.

A remarkable difference with the syntax of English is that in Gaelic has no prototypical verbal passives, but different structures to denote a prominent role of the patient over the agent. For example a construction with the auxiliary–*rach* with the meaning of “come to pass,” mostly used with transitive verbs. Another option is the combination of an aspectual marker *air* + verb “to be.” As in the sentence *Bha an rìgh air a mharbhadh be.PAST* (“The king AIR his kill VN,” “The king was killed”).

Scottish Gaelic, as a language spoken by few people, is influenced by the dominant language, English. A noticeable trend in the syntax of Gaelic exemplifying the impact of English, is the tendency for younger speakers to answer to a Yes/No to with *tha* and *chan eil*. ScG does not have a lexical affirmation/negation term for polar questions, with the most typical answer being the repetition of the verb preceded by cha(n). An answer such as tha and chan eil is viewed as ungrammatical for a traditional Gaelic speaker.

### Language and Cognition in Bilingualism With Minority Languages

Over the last 15 years, research has focused both on the linguistic and the general cognitive effects of bilingualism (Bialystok, 2009; Baum and Titone, 2014). Many studies (but not all, see Paap and Greenberg, 2013) have found a positive effect of bilingualism on language development (Prior and MacWhinney, 2010; Bartolotti and Marian, 2012; Marian and Shook, 2012) and general cognitive functions, such as the control of attention. However, there is a limited number of studies on the cognitive effects of bilingualism in minority languages, and their results are mixed. While some have found no evidence of a cognitive advantage in minority language bilinguals (Gathercole et al., 2014 for Welsh-English; Duñabeitia et al., 2014 for Basque-Spanish), others did find an advantage in terms of cognitive control in selective tasks, for example in working memory (Antoniou et al., 2014 for Greek-Cypriot Greek; Lauchlan et al., 2013 for a comparison of Gaelic-English with Sardinian-Italian; Vangsnes et al., 2017 for a study of biliteracy in Nynorsk and Bokmal) or for selective grammatical structures such as object relative clauses (Garraffa et al., 2015, 2017). Garraffa et al. (2015) research is particularly relevant for the purpose of our study. It looks at Sardinian-Italian bilingual children in two different age groups (at the beginning of primary school and after 1 year of primary education) living in an area were Sardinian is widely spoken, and compares their cognitive and linguistic skills with monolingual Italian children’s living in the same area. Results showed that across age groups, the performance of Sardinian–Italian bilingual children on the dominant language was in most cases indistinguishable from that of monolingual Italian children, in terms of both Italian language skills and general cognitive abilities. In some cases, however, there was a difference in favor of the older bilingual children, which was more pronounced for complex sentences, such as center-embedded relative clauses, such as *The mum who the child is kissing is blonde*. This implies that being bilingual in a minority language does not negatively affect the community language either cognitively or in terms of language proficiency. However, the positive cognitive effects of bilingualism that were found seemed to emerge gradually over time.

Overall, the results from these studies are difficult to compare, as minority languages typically receive varying levels of institutional support (e.g., Welsh vs. ScG) and the number of speakers varies, affecting background measures. The diversity of contexts in which minority languages are used points to the importance of understanding the influence of factors such as level of education, typological distance from the dominant language, and patterns of language use, and stresses the need for more investigations on bilingual language development in different circumstances.

### New Speakers

In previous research, new speakers have been investigated under various labels, e.g., “non-native speaker,” “second language speaker,” “L2 speaker,” and “learner.” For the sake of clarity, in the current study we use the term “new speaker” as defined by McLeod and O’Rourke (2015), “people who did not acquire Gaelic in the home when growing up, but have nevertheless acquired a significant degree of competence in the language and are now making active use of the language in their lives.” Using this label enables us to distance ourselves from the native/non-native speaker dichotomy, and to include different language learning trajectories in the debate on bilingualism (O’Rourke and Pujolar, 2013). By contrast, the term “native speakers” refers to “*learners of the language who had extensive exposure to Gaelic while growing up but did not acquire active competence in it*” (ibid). In the context of language revitalisation and maintenance policies, the number of native speakers is declining, while that of new speakers is increasing, as with other minority languages (see O’Rourke and Ramallo, 2013 for Galician; O’Rourke and Walsh, 2015, 2020 for Irish; Hornsby, 2008 for Breton; Price and Tamburelli, 2016 for Welsh; Ortega et al., 2015 for Basque). The resulting diversity of speakers may subsequently generate tensions between communities, especially in terms of authenticity, ownership and legitimacy of the language (O’Rourke and Ramallo, 2013; McLeod and O’Rourke, 2015; O’Rourke and Pujolar, 2015). It is precisely these issues that have been the focus of research on speakers of minority languages, and especially the status and role of new speakers. However, most studies investigate either children attending medium education programs and in the process of learning the language, or adults, especially those using Gaelic in the workplace. Common findings about adults’ perceptions and experiences across studies can be summarized as follows: (a) adult new speakers often consider native speakers as a model of “good Gaelic;” (b) they tend to consider their Gaelic as less “pure” and worthy; (c) they are more willing to use Gaelic frequently and in different environments than native speakers; (d) yet, native speakers do not always value their efforts and sometimes switch to English; (e) the Gaelic spoken by Glaswegian and Edinburgh adult new speakers is sometimes perceived as an “urban construct” (McLeod and O’Rourke, 2015), due to speakers’ urban accents; lastly, (f) there are too few opportunities to use Gaelic in the public sphere, enhancing the sense of illegitimacy when it comes to using Gaelic in a wider context. In the context of GME, the majority of the students are New speakers, with no opportunity to practice the language at home and no exposure to the language in early years. In this study we will look at the performance of the New speakers compared to Home speakers considering if the consistent school input in the minority language will supersede the lack of input in the home environment.

### Research Questions

In the context of the debate of the effects of bilingualism with a minority language, our study aimed to achieve four main goals. First, we wanted to test the hypothesis of an effect of bilingualism on language proficiency by measuring grammatical abilities in English monolingual children and bilingual children attending an immersion school on Gaelic. Second, we looked at the effect of language exposure on both linguistic and cognitive abilities, comparing Home Speakers (speakers who learned the minority language at home) and New Speakers (speakers who started learning the language in the school and with no Gaelic at home). Third, we examined the relationship between grammatical abilities in complex sentences and cognitive tasks targeting interference of pragmatic abilities, looking at possible interactions between cognition and comprehension of center-embedded and relative clauses.

Our fourth and final goal was to contribute on the debate regarding revitalization of minority languages, looking at overall differences in grammatical abilities and cognition in young adults, and comparing the effects of language immersion programs in areas where the medium school represents the only opportunity to speak the language in the community.

The main aim of the study was to explore the linguistic and cognitive profile of young adults learning Gaelic through the school medium and compare abilities of home speakers, new speakers and monolinguals at the end of the school cycle (14-years education in the language). We address the following questions:

Is there a difference between the linguistic English skills of monolingual speakers of English and Gaelic/English home young adult speakers of Gaelic, and new speakers of Gaelic attending GME?

Is there a difference between the cognitive skills of Home speakers of Gaelic and New speakers of Gaelic (both attending GME), in particular with respect to working memory and attentional control?

Is there an interaction between comprehension of complex sentences and control of interference in general attentional task?

Are the possible effects on language and cognition due to the higher socio-economic background of the bilingual young adults?

## Materials and Methods

### Participants

Participants were recruited from one secondary school with English as medium for education and one secondary school providing immersion education in Gaelic from nursery through the end of the secondary program (from 3 years of age up to 18). They were all young adults aged 16–18 years (mean = 16.4, SD = 0.58), attending S5 and S6, i.e., the last 2 years of Secondary Education in Scotland. Academic performance in English were reported as typical for their stage level. Students with no standard scores and beyond in their exams results were not invited to take part in the study, Number of students excluded was not shared. Parents of those willing to participate returned information and consent sheets and a questionnaire indicating whether they were exposed to Gaelic at home. 49 participants took part to the study, one participant was excluded as they performed below standard scores for their age band in the standard test, leaving 48, of which 23 were from the monolingual school (age mean 16.6, SD 0.58. In the two bilingual groups, 10 participants were exposed to Gaelic from birth, age mean 16.1 SD 0.32 (henceforth “home speakers;” male = 6, female = 4) and 15 were first exposed to Gaelic through nursery, age-mean 16.4 SD 0.63 (henceforth “new speakers;” male = 3, female = 12).

Students attending the monolingual school were all resident in the catchment area of the school. All postcodes indicated an area considered not deprived (decile 10, quintile 5 of the Scottish of Multiple deprivation index, SIMD)^1^. Students attending the bilingual GME schools had a different set of postcodes, with many of them based in the most deprived area (decile 1, quintile 1).

### Language Profiles

Bilingual participants completed the Bilingual Language Profile (BLP; Birdsong et al., 2012), supplemented by two questions about accent perception from the Language Experience and Proficiency Questionnaire (LEAP-Q; Marian et al., 2007). The BLP consists of 17 questions in total, divided into four categories, i.e., *Language History*, *Language Use*, *Language Proficiency*, and *Language Attitudes*. Participants in the two bilingual groups, home speakers and new speakers, did not differ on any of the BLP factors excluding Language History, where there was a significant difference in the number of years they had spent in a family where Gaelic is used: 15 years average for Home speakers (SD 4.84) and 9 years for new speakers (SD 7.83). No other differences were found in the other categories.

### Experimental Measures

#### Linguistic Measures

All participants took the Test for the Reception of Grammar, version 2 (TROG-2; Bishop, 2003) to measure their comprehension in English. This test consists of 20 blocks of four utterances each, with each block testing different grammatical structures, starting from simple two elements active sentences to more complex center-embedded clauses (see Table 1 for the full list of TROG-2 blocks and one sentence example). In particular, according to the test norms, we are expecting some differences in performance on the more complex blocks and at ceiling performance with simpler sentences.

**TABLE 1:** Example of structures from the TROG-2 (Bishop, 2003).

Grammatical structures	Example
Relative clause in object	The girl chases the dog that is jumping.
Reversible subject-verb order	The cat is looking at the boy.
Relative clause in subject	The man that is eating looks at the cat.
Center-embedded sentence	The sheep the girl looks at is running.
Reversible passive	The cow was chased by the girl.
Pronoun binding	The man sees that the boy is pointing at him.

**FIGURE 2:**
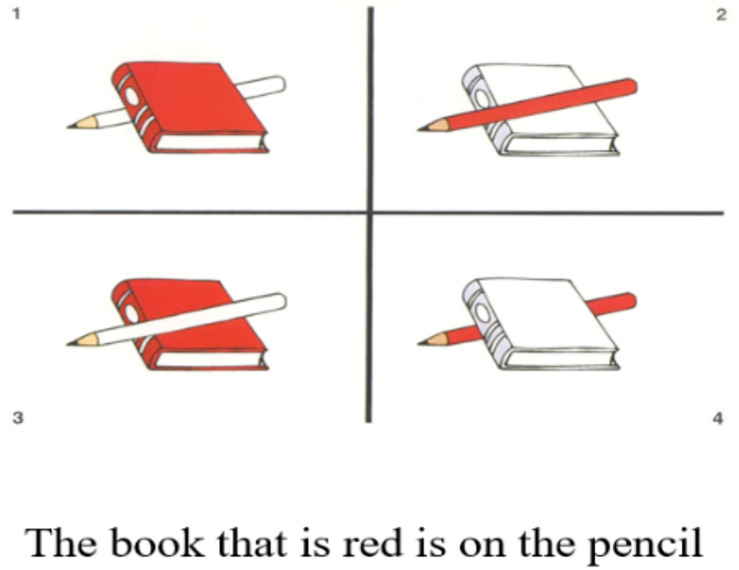
Example of a picture-sentence matching task targeting a relative clause, TROG- 2 (Bishop, 2003).

The test was based on a booklet of stimulus pictures, with four pictures for each sentence and the target utterance at the bottom, as shown in Figure 2. Participants listened to a sentence read aloud by the experimenter and had to match it with the picture they thought corresponded to the sentence. The test took approximately 20 min and the experimenter scored each utterance manually on the basis of the provided scoring sheet. To pass a block, participants had to match all four items in the block correctly.

#### Cognitive Measures

##### Attention

Participants took the Test of Everyday Attention, version 2 (TEA-2; Robertson et al., 1994). This is a widely used clinical test assessing several aspects of attentional control, which has recently been adapted for use in research on typical bilingualism (Bak et al., 2016). It consists of three standardized subsets, i.e., the *Elevator Task*, *the Elevator Task with Distraction*, and the *Elevator Task with Reversal*. Each of these subsets lasts approximately 5 min and the experimenter scores each test manually on a scoring sheet. In the *Elevator Task*, which tests auditory sustained attention, participants heard a series of pre-recorded low tones occurring at varying intervals and were asked to count them. The *Elevator Task with Distraction* tests targets auditory selective attention and inhibitory control. During the test, participants heard a series of low tones and high tones at varying intervals, and were asked to count only the low tones, while disregarding the high tones. The third subtest, the *Elevator Task with Reversal*, targets inhibitory control and switching abilities, including noticing a cue that prompts the refocusing of attention. Participants heard high, middle and low tones. They had to count the middle tones upward when preceded by a high tone (excluding the high tone), and to count them downward when preceded by a low tone (excluding the low tone). The high and low tones only signaled the directionality of counting, i.e., whether the participants needed to add (high tone) or subtract (low tone) the middle tones. Participants sat in front of a computer screen and the experimenter played each subset of recordings. There was a practice session before each subset.

##### Working memory

Participants also performed the Backward Digit Span test of working memory (WAIS-III, Wechsler, 1991). The experimenter read a sequence of digits, one second per digit, and then the participant had to repeat the sequence backward. Each sequence was incrementally longer (3 digits, 4 digits, 5 digits, and so on up to 8 digits). A sequence was considered correct if the participant repeated the whole sequence in the correct reverse order. There were two sequences for each digit length. A score consisted of the longest sequence repeated correctly, with a score of two when correct on first trial, and 1 when correct on second trial.

All the background and experimental tests were administered in one session of approximately 45 min, which took place in a quiet room in the school.

## Results

The following analyses considered differences between monolingual and bilingual groups, as well as between the three study groups (*Monolinguals*, *Home speakers*, and *New speakers*). Consequently, the following descriptive statistics have summaries for all these groupings.

### Linguistic Measures

Three measures from the TROG-2 were used to compare linguistic abilities between groups, namely, Total raw score, Relative clauses accuracy (blocks G, S, and T) and more in detail the score on the more complex Center-Embedded clauses (Block T, Figure 2). Total standardized scores are reported in the descriptive tables to facilitate comparisons with other studies. These linguistic descriptive measures are shown in Table 2.

**TABLE 2:** Descriptive statistics for SIMD (0–20) and TROG-2 for monolingual and bilingual groups: Total Standardized score; Relative clause correctness (0–4, but actual range was 3–4) and Center-Embedded clause correctness (0–4).

Languages	Bilinguals	*N*	Age mean (sd)	SIMD median (iqr)	TROG-2 Std mean (sd)	Relative Clause median (iqr)	Center-Embedded median (iqr)
Monolingual		23	16.6 (0.58)	3.0 (1.00)	92.9 (7.39)	3.0 (0.00)	2.0 (1.00)
Bilingual		25	16.3 (0.54)	9.0 (10.00)	94.1 (8.87)	4.0 (1.00)	3.0 (1.00)
	Home Speakers	10	16.1 (0.32)	15.5 (8.75)	93.2 (8.22)	4.0 (1.00)	2.0 (1.75)
	New Speakers	15	16.4 (0.63)	7.0 (9.00)	94.7 (9.51)	4.0 (0.50)	3.0 (1.50)

*Values shown are either mean (standard deviation), or median (inter-quartile range).*

The Scottish Index of Multiple deprivation (SIMD) median scores for each group are also shown in Table 2. It is interesting to note that while socio-economic status measured by means of the SIMD varied between groups. However, children from more deprived areas and children with a higher family income performed similarly on the standardized TROG-2.

### Cognitive Measures

Backward digit span and TEA were used as markers of cognitive ability in the three different groups. Table 3 shows median and inter-quartile ranges for backward digit span and for the three TEA measures. Median and IQR were used as measures of central tendency as these parameters do not have a normal distribution. Working memory (as measured by Backward digit span) does not differ significantly between the between the monolinguals and the two bilingual groups.

**TABLE 3:** Descriptive statistics for cognitive measures: Backward digit span (0–8); Test of Everyday Attention-1, TEA-1 (0–7); TEA-2 (0–10); TEA-3 (0–10).

Languages	Bilinguals	*N*	TEA-1 median (iqr)	TEA-2 median (iqr)	TEA-3 median (iqr)	Backward Digit Span median (iqr)
Monolingual		23	7.0 (1.00)	8.0 (3.50)	5.0 (2.50)	6.0 (1.50)
Bilingual		25	7.0 (0.00)	9.0 (2.00)	8.0 (5.00)	6.0 (1.00)
	Home Speakers	10	7.0 (0.00)	9.5 (2.00)	8.5 (2.75)	6.5 (1.00)
	New Speakers	15	7.0 (0.00)	9.0 (2.00)	8.0 (4.50)	6.0 (1.50)

However, the more complicated TEA-2 and TEA-3 do show the monolingual group to be slightly suppressed when compared to the combined bilingual group. The distributions for these tests are non-normal and constrained between 0 and 7 (TEA-1) or 0 and 10 (TEA-2 and TEA-3).

### Regression Modeling

Language comprehension was explored with the TROG-2 total raw score as the dependent variable with Gaussian error distribution regression models. Variables for cognitive function are limited to small ranges of possible values, hence regression models that assume Gaussian distributions are inappropriate for these measures. We therefore used generalized regression models with *Poisson* distributions that are appropriate for discrete data ranges where the cognitive measures were the dependent variable.

To address the question of whether the data supported generalized regression modeling, a power analysis was performed for TROG-2. Taking R^2^ from the second regression model in Table 4 [TROG-2 ∼ Monolingual vs. Bilingual + Age, with df = (2,45)] to estimate effect size, the resulting power for the generalized regression model was 0.848 at the 0.05 level. When all three test groups were considered (model 3 in Table 4), the resulting power for the regression was 0.870 (df = (3,44), sig. level = 0.05). These power levels are satisfactory for the generalized linear regression modeling used in this analysis.

**TABLE 4:** Regression models (lm()) of TROG-2 total score by Language groups (either Monolingual/Bilingual, or Monolingual/Home Speakers/New Speakers).

	Dependent variable:		
	TROG-2 total raw score		
	(1)	(2)	(3)
(Intcpt)/	73.688	72.315	72.353
2,3:Monolingual	*t* = 149.898	*t* = 106.359	*t* = 107.308
	*p* = 0.000	*p* = 0.000	*p* = 0.000
2: Bilingual		2.635	
		*t* = 2.741	
		*p* = 0.009	
3: Home speakers			1.493
			*t* = 1.175
			*p* = 0.247
3: New Speakers			3.274
			*t* = 3.080
			*p* = 0.004
Age	−0.860	−0.479	−0.607
	*t* = –1.731	*t* = –0.988	*t* = –1.239
	*p* = 0.091	*p* = 0.329	*p* = 0.222
Observations	48	48	48
*R*^2^	0.061	0.195	0.228
Adjusted *R*^2^	0.041	0.160	0.175
Residual Std. Error	3.406 (df = 46)	3.188 (df = 45)	3.158 (df = 44)
F Statistic	2.995* (df = 1; 46)	5.466*** (df = 2; 45)	4.324*** (df = 3; 44)

Model 1: TROG-2 ∼ Age. Model 2: TROG-2 ∼ Monoling/Biling + Age. Model 3: TROG-2 ∼ Monoling/Home Speakers/New Speakers + Age. *p ¡ 0.1; **p ¡ 0.05; ***p ¡ 0.01.

#### Modeling Language Proficiency by Language Groups

Regarding our first research question concerning linguistic skills between the monolingual and bilingual groups, and between all three language groups, we carried out linear regressions of these groups onto TROG-2 total raw score. Table 4 reports three regression models in hierarchical (embedded) regression style.

The first model (the base model) uses Age as a fixed effect predictor as it significantly explains some variance around TROG-2 (coeff = −0.860, *t*(df = 48) = −1.731, *p* = 0.091). Interestingly, the coefficient is negative implying that greater scores on the TROG-2 associate with decreasing ages.

The second regression model in Table 4 adds a Monolingual/Bilingual fixed predictor to model 1. As can be seen from the model statistics, this second model improves upon the first model (*R*^2^ = 0.061 for model 1, and *R*^2^ = 0.195 for model 2). Moreover, the Bilingual coefficient is 2.635 (*p* = 0.009) meaning that bilinguals (Home speakers plus New speakers) significantly outperform monolinguals on the TROG-2.

The third model in Table 4 further breaks down the language into the three experiment groups, namely monolingual, home speakers and new speakers. Again, this model explains more variance than model 2 (model 3 *R*^2^ = 0.228). However, the comparison between language groups shows that while New speakers significantly outperform monolinguals (New Speakers coeff = 3.274, *p* = 0.004), Home speakers are not significantly differentiated from monolinguals (Home speakers *t* = 1.175, *p* = 0.247). In both models 2 and 3, Age does not explain a significant amount of variance around TROG-2. This could be that there is an association between Age and language groupings (c.f. Ages in Table 2).

#### Modeling Cognitive Skills by Language Groups

The second research question addresses working memory and attentional control, asking if the different language groups perform differently on these measures. Table 5 (Home speakers vs. New speakers) and Table 6 (Monolingual vs. Bilingual) show these regression models. As these dependent measures are finite (0–7, for TEA-1; 0–10 for TEA-2 and 0–8 for backward digit span), we used general linear regression with *Poisson error* distribution for a finite count dependent variable. The four cognitive measures used in this study can be considered as finite count variables.

**TABLE 5:** Regression model for cognitive abilities (TEA-1, TEA-2, TEA-3, and Backward digit Span) by bilinguals (Home speakers vs. New Speakers).

	Dependent variable:			
	TEA-1	TEA-2	TEA-3	Backward digit span
	(1)	(2)	(3)	(4)
(Intcpt)/Home	1.946	2.104	1.988	1.841
Speakers	*t* = 16.281	*t* = 19.054	*t* = 16.984	*t* = 14.609
	*p* = 0.000	*p* = 0.000	*p* = 0.000	*p* = 0.000
New speakers	−0.039	0.040	−0.052	−0.016
	*t* = −0.250	*t* = 0.282	*t* = −0.338	*t* = −0.098
	*p* = 0.803	*p* = 0.779	*p* = 0.736	*p* = 0.922
Observations	25	25	25	25
Log Likelihood	−47.672	−57.761	−72.642	−48.789
Akaike Inf. Crit.	99.343	119.523	149.284	101.579

*The glm() function was used with a *Poisson* error distribution. Model 1: TEA-1 ∼ Home Speakers/New Speakers, Poisson error dist. Model 2: TEA-2 ∼ Home Speakers/New Speakers, Poisson error dist. Model 3: TEA-3 ∼ Home Speakers/New Speakers, Poisson error dist. Model 4: Backward digit span ∼ Home Speakers/New Speakers, Poisson error dist.*

**TABLE 6:** Regression modeling of cognitive measures by language groups (Monolingual vs. Bilingual).

	Dependent variable:			
	TEA-1	TEA-2	TEA-3	Backward digit span
	(1)	(2)	(3)	(4)
(Intcpt)/	1.875	1.921	1.685	1.732
Monolingual	*t* = 22.966	*t* = 24.067	*t* = 18.761	*t* = 19.748
	*p* = 0.000	*p* = 0.000	*p* = 0.000	*p* = 0.000
Bilingual	0.048	0.207	0.272	0.099
	*t* = 0.426	*t* = 1.967	*t* = 2.327	*t* = 0.833
	*p* = 0.671	*p* = 0.050	*p* = 0.020	*p* = 0.405
Observations	48	48	48	48
Log Likelihood	−91.414	−109.165	−132.148	−93.658
Akaike Inf. Crit.	186.827	222.331	268.296	191.316

*The glm() function was used with a *Poisson* error distribution. Model 1: TEA-1 ∼ Monolingual/Bilingual, Poisson error dist. Model 2: TEA-2 ∼ Monolingual/Bilingual, Poisson error dist. Model 3: TEA-3 ∼ Monolingual/Bilingual, Poisson error dist. Model 4: Backward digit span ∼ Monolingual/Bilingual, Poisson error dist.*

In Table 5 TEA-1, TEA-2, TEA-3 and backward digit span are modeled by the two bilingual groups. None of these models differentiate between Home speakers and New speakers on these cognitive measures (*t* = −0.25 for TEA-1; *t* = 0.282 for TEA-2; *t* = −0.338 for TEA-3; *t* = −0.098 for backward digit span).

Cognitive performance modeled by Monolingual vs. Bilingual groups on the other hand does show that the more complex TEA-2 and TEA-3 do differ between groups. In Table 6, TEA-2 bilingual coeff = 1.967 (*p* = 0.05) and TEA-3 bilingual coeff = 2.327 (*p* = 0.02), implying that bilinguals outperform monolinguals on these attentional measures. Interestingly, neither backward digit span nor TEA-1 differ between groups (*t* = 0.833 and *t* = 0.426, respectively). As explained above, the three tests for everyday attention are incrementally more complex implying that the bilingual groups (undifferentiated between themselves, Table 5) have an advantage over monolinguals with incremental attentional task challenge.

#### Modeling Cognitive Measures by Experiment Groups and Linguistic Complexity

The next research question concerns whether the bilingual advantage identified in Table 6 interacts with linguistic complexity. For this, we use the relative clauses (Table 7) and center-embedded (Table 8) components of the TROG-2 as proxies for linguistic complexity. In these models the linguistic variable is purposefully added as an interaction with group shown in Table 6. Any improvement in accounting for variance around the dependent measure will be reflected in a significantly lower Log Likelihood for the regression model.

**TABLE 7:** Does comprehension of relative clause structure interact with bilingualism for cognitive function? glm() regression with *Poisson* error were used to model cognitive parameters.

	Dependent variable:			
	TEA-1	TEA-2	TEA-3	Backward digit span
	(1)	(2)	(3)	(4)
(Intcpt)/Monolingual	1.897	1.917	1.660	1.692
	*t* = 19.539	*t* = 19.646	*t* = 14.784	*t* = 15.165
	*p* = 0.000	*p* = 0.000	*p* = 0.000	*p* = 0.000
Bilingual	0.021	0.239	0.322	0.132
	*t* = 0.159	*t* = 1.939	*t* = 2.312	*t* = 0.920
	*p* = 0.874	*p* = 0.053	*p* = 0.021	*p* = 0.358
Relative-Clause	0.043	−0.007	−0.045	−0.073
	*t* = 0.412	*t* = −0.064	*t* = −0.371	*t* = −0.603
	*p* = 0.681	*p* = 0.949	*p* = 0.711	*p* = 0.547
Bilingual: Rel-Clause	−0.034	−0.055	−0.010	0.087
(Interaction)	*t* = −0.251	*t* = −0.432	*t* = −0.071	*t* = 0.583
	*p* = 0.802	*p* = 0.666	*p* = 0.944	*p* = 0.560
Observations	48	48	48	48
Log Likelihood	−91.323	−108.801	−131.834	−93.458
Akaike Inf. Crit.	190.647	225.601	271.668	194.915

*Model 1: TEA-1 ∼ Monoling/Biling × Relative Clause, Poisson error. Model 2: TEA-2 ∼ Monoling/Biling × Relative Clause, Poisson error. Model 3: TEA-3 ∼ Monoling/Biling × Relative Clause, Poisson error. Model 4: Backward digit span ∼ Monoling/Biling × Relative Clause, Poisson error.*

**TABLE 8:** Does comprehension of center-embedded clauses interact with bilingualism for cognitive function? glm() regression with *Poisson* error were used to model cognitive parameters.

	Dependent variable:			
	TEA-1	TEA-2	TEA-3	Backward digit span
	(1)	(2)	(3)	(4)
(Intcpt)/Monolingual	1.866	1.881	1.621	1.705
	*t* = 20.389	*t* = 20.545	*t* = 15.451	*t* = 17.105
	*p* = 0.000	*p* = 0.000	*p* = 0.000	*p* = 0.000
Bilingual	0.062	0.215	0.241	0.093
	*t* = 0.507	*t* = 1.815	*t* = 1.772	*t* = 0.700
	*p* = 0.612	*p* = 0.070	*p* = 0.077	*p* = 0.485
Center-Embedded	−0.025	−0.099	−0.152	−0.069
	*t* = −0.235	*t* = −0.947	*t* = −1.299	*t* = −0.605
	*p* = 0.815	*p* = 0.344	*p* = 0.194	*p* = 0.546
Bilingual:	0.011	0.182	0.362	0.155
Center-Embedded	*t* = 0.088	*t* = 1.468	*t* = 2.593	*t* = 1.122
(Interaction)	*p* = 0.930	*p* = 0.143	*p* = 0.010	*p* = 0.262
Observations	48	48	48	48
Log Likelihood	−91.369	−107.938	−127.327	−92.858
Akaike Inf. Crit.	190.738	223.875	262.654	193.716

*Model 1: TEA-1 ∼ Monoling/Biling × Center-Embedded, Poisson error. Model 2: TEA-2 ∼ Monoling/Biling × Center-Embedded, Poisson error. Model 3: TEA-3 ∼ Monoling/Biling × Center-Embedded, Poisson error. Model 4: Backward digit span ∼ Monoling/Biling × Center-Embedded, Poisson error.*

As can be seen by comparing the Log Likelihood values between Tables 6, 7 shows that relative clauses do not interact with the bilingual advantage seen in Table 6. Log likelihood = −109.165 for the TEA-2 model in Table 6 vs., log likelihood = −108.801 for the TEA-2 model in Table 7; and −132.148 vs. −131.834 for the TEA-3 models in Tables 6, 7. Both of these log likelihood differences are non-significant (anova Chi Square *p* = 0.694 for the TEA-2 models and *p* = 0.731 for the TEA3 models).

Table 8 shows the models with center-embedded added as an interaction term with groups to the cognitive models in Table 6. Interestingly the TEA-3 models differ (Log likelihood = −132.148 for TEA-3 modeled by Monolingual vs. Bilingual, Table 6; Log likelihood = −127.327 for TEA-3 modeled by Monolingual/Bilingual interacting with Center-Embedded, Table 8; anova Chi Square *p* = 0.008 for these two models). The Bilingual:Center-Embedfed interaction term has a positive coeff = 0.362 (*p* = 0.01) meaning that bilinguals are performing even better than expected on the center-embedded tasks.

#### Does SIMD Associate With Experiment Groups or Cognition Measures?

Since there are SIMD differences between groups (see Table 2), the next question is whether socioeconomic circumstances could be driving the bilingual advantages identified in model 3, Table 4, or models 2 and 3, Table 6.

Table 9 repeats the models in Table 4 with SIMD added as an interaction term. As can be seen, non of the interaction terms (Bilingual:SIMD or New Speaker:SIMD) are significant (*p* = 0.828 and *p* = 0.479, respectively).

**TABLE 9:** Regression modeling for possible SIMD interaction with bilingualism in language comprehension measured with TROG-2.

	Dependent variable:		
	TROG-2 total raw score		
	(1)	(2)	(3)
(Intcpt)/2:Monoling/	73.688	72.290	75.027
3:HomeSpk	*t* = 148.789	*t* = 40.845	*t* = 45.069
	*p* = 0.000	*p* = 0.000	*p* = 0.000
2: Bilingual		3.024	
		*t* = 1.570	
		*p* = 0.124	
3: New Speakers			0.551
			*t* = 0.293
			*p* = 0.773
SIMD	0.754	0.173	−0.720
	*t* = 1.507	*t* = 0.071	*t* = −0.634
	*p* = 0.139	*p* = 0.944	*p* = 0.533
2: Biling : SIMD		−0.552	
(interaction)		*t* = –0.219	
		*p* = 0.828	
3: New Speaker : SIMD			1.059
(interaction)			*t* = 0.721
			*p* = 0.479
Observations	48	48	25
*R*^2^	0.047	0.185	0.076
Adjusted *R*^2^	0.026	0.129	−0.055
Residual Std. Error	3.431 (df = 46)	3.245 (df = 44)	3.275 (df = 21)
F Statistic	2.273 (df = 1; 46)	3.319** (df = 3; 44)	0.580 (df = 3; 21)

Model 1: TROG-2 ∼ SIMD. Model 2: TROG-2 ∼ Monoling/Biling × SIMD. Model 3: TROG-2 ∼ Home Speakers/New Speakers × SIMD. *p ¡ 0.1; **p ¡ 0.05; ***p ¡ 0.01.

Table 10 repeats the models in Table 6 with SIMD added as an interaction term. The Bilingual:SIMD interation term for the TEA-2 and TEA-3 models are non-significant as well (*p* = 0.598 and *p* = 0.3, respectively). Thus, the bilingual advantages in TROG-2 and in TEA-2 and TEA-3 are not driven by group differences in SIMD.

**TABLE 10:** Regression modeling for possible SIMD interaction with bilingualism in cognitive function. glm() regression with *Poisson* error distribution was used.

	Dependent variable:			
	TEA-1	TEA-2	TEA-3	Backward digit span
	(1)	(2)	(3)	(4)
(Intcpt)/Monolingual	1.820	1.835	1.457	1.654
	*t* = 8.293	*t* = 8.430	*t* = 5.562	*t* = 6.942
	*p* = 0.000	*p* = 0.000	*p* = 0.00000	*p* = 0.000
Bilingual	0.104	0.271	0.474	0.174
	*t* = 0.437	*t* = 1.166	*t* = 1.713	*t* = 0.677
	*p* = 0.663	*p* = 0.244	*p* = 0.087	*p* = 0.499
SIMD	−0.082	−0.127	−0.335	−0.115
	*t* = −0.272	*t* = −0.426	*t* = −0.943	*t* = −0.353
	*p* = 0.786	*p* = 0.671	*p* = 0.346	*p* = 0.725
Bilingual: SIMD	0.081	0.161	0.376	0.120
(Interaction)	*t* = 0.261	*t* = 0.527	*t* = 1.038	*t* = 0.357
	*p* = 0.795	*p* = 0.598	*p* = 0.300	*p* = 0.721
Observations	48	48	48	48
Log Likelihood	−91.376	−108.947	−131.520	−93.593
Akaike Inf. Crit.	190.752	225.893	271.040	195.185

*Model 1: TEA-1 ∼ Monoling/Biling × SIMD, Poisson error. Model 2: TEA-2 ∼ Monoling/Biling × SIMD, Poisson error. Model 3: TEA-3 ∼ Monoling/Biling × SIMD, Poisson error. Model 4: Backward digit span ∼ Monoling/Biling × SIMD, Poisson error.*

## Discussion

This study sought to investigate Gaelic-English young adults’ linguistic and cognitive abilities. The aim was to bridge a gap in the literature by focusing on young adults–an age range not considered by previous research–and comparing Home Gaelic speakers New Gaelic speakers. The study should be consider as a pilot, due to the small number of participants. There is a need of a bigger sample in future study tracking in detail language and cognition in minority language speakers learning a language via an immersion program.

Investigating linguistic and cognitive abilities in the minority language is crucial in order to understand and assess new speakers’ language maintenance and use of Gaelic in a wider range of contexts than the classroom. The discussion will summarize our results and address a few limitations of our study.

### Language Proficiency

The first result was the higher scores compared to monolinguals on the English sentences comprehension task (TROG-2), despite the dominance of Gaelic since nursery and in primary school. Crucially, this study confirms previous findings on minority languages (e.g., Garraffa et al., 2015 on Sardinian-Italian bilinguals), as it further shows that full immersion in a second language since nursery does not negatively affect linguistic competence in the majority language. If a difference is reported this is in favor of the participants enrolled in a GME program. It is interesting to note that a similar performance was reported in the school qualifications English exams, with students from GME schools performing at the top of the list compared to other secondary schools in the country^2^. A in-depth investigation of the sentence comprehension performance revealed not only that young adults in GME outperform monolinguals but also that among the GME group there seems to be a more marked advantage for New Speakers with respect to the more complex center-embedded clauses compared to both monolinguals and Home Speakers. This is a result that needs further investigation and a closer look at the community of New speakers in this particular ScG context. One could speculate that being an Home speaker does not offer any clear advantage compared to the New speaker, as it has been reported that Home speakers have English as the only family language and tend not to use Gaelic at home. Together with the lack of a bilingual environment at home for the Home speakers of Gaelic, it is possible that many of the New speakers of Gaelic do live in a multilingual family, with a richer context to practice language switching and cognitive inhibition.

### Cognition

The second question addressed was whether there would be any difference between the cognitive skills of Home and New speakers of Gaelic. As expected, we found that both Home speakers and New speakers performed at ceiling in the tests of attentional control. Young adult bilinguals in this age range usually perform at ceiling on measures of executive functions, since they are at the peak of their cognitive abilities (Bialystok et al., 2004). In addition, both groups consisted of bilinguals, who have often been reported to have an advantage in terms of cognitive control. Moreover, the similar results in the two groups are further strengthened by the fact that they have similar patterns of use of Gaelic and have been following the same immersion program since nursery.

More interesting were the results of our models in relation to the interactions of bilinguals with TEA-3 and the variance for center-embedded correctness. Similarly to what reported in other studies investigating an interaction between working memory and center-embedded clauses (Lauro et al., 2010), our population and in particular the bilingual group reported an interaction between comprehension of center-embedded relatives and attentional control, in particular the *Elevator Task with Reversal* that targets inhibitory control and switching abilities, including noticing a cue that prompts the refocusing of attention (TEA-3). A possible speculation is that these two tasks, comprehension of a center-embedded clause and the inhibition of an active element as in the TEA-3, shared a cognitive resource enhanced in bilingual speakers. It is well know from other studies on language development and pathologies that Center-embedded relative clause, such as *The man the elephant sees is eating*, are selectively modulated by the presence of an active intervener and the similarity between the two nouns (see Garraffa and Grillo, 2008 and Martini et al., 2019 for adults with pathologies; Garraffa et al., 2017 for bilingual speakers). In these sentences the subject noun, *The man*, is holded in memory before its verb, *is eating*, will be integrated. During this simple memory task, another noun, *the elephant*, needs to be also holded in memory. This operation of holding two potential arguments for the main verb is very costly and this was reported to be particularly taxing if both nouns are grammatically similar (for example both are singular or animate). The second noun, the elephant, is interfering while the subject noun is holded in memory, causing many reversible errors with participants selecting a picture where is the elephant eating and not the man. Bilingual speakers are less prone to this cost with a lower disruptive effect of the intervener element, possibly due to a more skilled control system in continuous alert for inhibition of active distractors.

More research is required to refine this hypothesis.

## Conclusion

This study investigated young Gaelic-English adult bilinguals and especially New speakers, who acquire the language in immersion education and have no exposure at home. This category represents the pillar of Gaelic maintenance and revitalisation. More specifically, the study focused on both linguistic and cognitive skills to investigate the interaction between grammatical knowledge and cognition. Not only did we find that both Gaelic Home Speakers’ and New Speakers’ cognitive and linguistic skills were comparable to those of Monolingual Speakers, but a positive correlation was found for complex structures and ability to focus in the context of an active distractor both on cognitive and linguistic tasks. Analyzing the Home Speakers and New Speakers’ language and cognitive experiences revealed that the dichotomy between home speakers and new speakers is very productive, as these two types of speakers have very similar linguistics and cognitive skills with a tendency for New Speakers to have more beneficial effects on cognition. All in all, these findings are encouraging as they show that GME is a good starting point to boost the number of Gaelic speakers, and that it does not negatively affect either their linguistic or cognitive skills. Combining cognitive and linguistic approaches is necessary to fully grasp new speakers’ experiences, but also to measure actual outcomes of the effects of medium education in different domains. More research adopting this comprehensive approach needs to be conducted for other minority languages, to be able to compare different contexts of acquisition and evaluate the outcome of policies aimed at revitalizing languages through active bilingualism.

## Data Availability Statement

The raw data supporting the conclusions of this article will be made available by the authors, without undue reservation.

## Ethics Statement

Ethical approval was obtained from both the Education Research Steering Group, Glasgow City Council and the Heriot-Watt School of Social Sciences Ethic Committee. Written informed consent to participate in this study was provided by the participants’ legal guardian/next of kin.

## Author Contributions

MG: conception and design of the work, analysis and interpretation of data, drafting the work and revising it critically for intellectual content, final approval of the version to be published, agreement to be accountable for all aspects of the work in ensuring that questions related to the accuracy or integrity of any part to the work are appropriately investigated and resolved. MO: analysis and interpretation of data for the work, drafting the work and revising it critically for intellectual content, agreement to be accountable for all aspects of the work in ensuring that questions related to the accuracy or integrity of any part of the work are appropriately investigated and resolved. BO’R: conception and design of the work, final approval of the version to be published, agreement to be accountable for all aspects of the work in ensuring that questions related to the accuracy or integrity of any part to the work are appropriately investigated and resolved. AS: conception and design of the work, interpretation of data for the work, drafting the work and revising it critically for important intellectual content, final approval of the version to be published, agreement to be accountable for all aspects of the work in ensuring that questions related to the accuracy or integrity of any part of the work are appropriately investigated and resolved. All authors contributed to the article and approved the submitted version.

## Conflict of Interest

The authors declare that the research was conducted in the absence of any commercial or financial relationships that could be construed as a potential conflict of interest.

## Footnotes

1 http://simd.scot/2016/#/simd2016/BTTTFTT/9/-4.0000/55.9000/

2 https://www.heraldscotland.com/news/17512100.pupils-who-learn-lessons-in-gaelic-come-top-of-the-class/
